# EHPnet: PAHO Communicable Disease Unit

**Published:** 2008-09

**Authors:** Erin E. Dooley

http://www.paho.org/english/ad/dpc/dpc-page.htm

The Pan American Health Organization (PAHO), an international public health agency, supports a Communicable Disease Unit (CD) whose primary functions include building networks and mobilizing resources for the prevention and control of communicable diseases; providing national and local training in areas such as clinical case management and use of standard guidelines for prevention and control activities; promoting and coordinating research; and developing policies, plans, and guidelines. Information collected by the CD is available through the top menu bar of PAHO’s Health Surveillance and Disease Prevention and Control website.

Besides information on diseases such as cholera, acute respiratory infections, and anthrax, the website provides information on mosquito-borne diseases such as dengue, malaria, West Nile virus, and yellow fever. Sections on each disease link to resources for disease surveillance, reports on the history of each disease, profiles of the disease in countries where it is occurring, and statistics on incidence and mortality. Each section also links to any available epidemiological surveillance systems and to laboratory networks established by PAHO for each disease. Visitors will also find resources for prevention, control, and educational efforts, including field guides, materials for families and communities, policy documents, overviews of global disease control strategies, travel advisories, journal articles, and bibliographic databases.

